# The Important Double-Edged Role of Astrocytes in Neurovascular Unit After Ischemic Stroke

**DOI:** 10.3389/fnagi.2022.833431

**Published:** 2022-04-07

**Authors:** Guangyuan Han, Lijuan Song, Zhibin Ding, Qing Wang, Yuqing Yan, Jianjun Huang, Cungen Ma

**Affiliations:** ^1^The Key Research Laboratory of Benefiting Qi for Acting Blood Circulation Method to Treat Multiple Sclerosis of State Administration of Traditional Chinese Medicine, Research Center of Neurobiology, Shanxi University of Chinese Medicine, Jinzhong, China; ^2^Department of Neurosurgery, Sinopharm Tongmei General Hospital, Datong, China; ^3^Department of Physiology, Shanxi Medical University, Taiyuan, China; ^4^Shanxi Key Laboratory of Inflammatory Neurodegenerative Diseases, Institute of Brain Science, Shanxi Datong University, Datong, China

**Keywords:** astrocytes, neurovascular units, ischemic stroke, BBB, double-edged role

## Abstract

In recent years, neurovascular unit (NVU) which is composed of neurons, astrocytes (Ast), microglia (MG), vascular cells and extracellular matrix (ECM), has become an attractive field in ischemic stroke. As the important component of NVU, Ast closely interacts with other constituents, which has been playing double-edged sword roles, beneficial or detrimental after ischemic stroke. Based on the pathophysiological changes, we evaluated some strategies for targeting Ast in treating ischemic stroke. The present review is focused on the roles of Ast in NVU and its complex signaling molecular network after ischemic stroke, which may be a prospective approach to the treatment of ischemic diseases in central nervous system.

## Introduction

Ischemic stroke, caused neurological dysfunction due to lack of blood supply to the brain with various reasons, has a high morbidity and mortality (Zhao et al., 2020). In the world, ischemic stroke has reached for nearly 87% of the total stroke patients, and continues to rise annually (Saini et al., 2021). At present, the therapy for ischemic stroke is mainly based on thrombolysis and neuroprotection, but none of them has achieved a satisfactory effect. Increasing clinical studies have shown that neurons, astrocytes (Ast), blood brain barrier (BBB) are suffering different degrees of damage after ischemic stroke, which may explain that targeting neurons or one of other cells seemly exerts almost no significant curative effect. The concept of a neurovascular unit (NVU) was proposed by American scientists Lo et al. (2003), which provided a systematical view on related comprehensive studies. Ast, as one of important NVU components, are accompanied with neurons, microglia (MG) and BBB.

In addition, recent studies have demonstrated the beneficial or detrimental roles of Ast in neurodegenerative diseases (Ding et al., 2021). Therefore, in this review, we will mainly discuss the function of Ast in NVU, and the role of Ast in NVU after ischemic stroke and strategies for targeting Ast in the treatment of ischemic stroke.

## The Functions of Astrocytes in Neurovascular Unit

**FIGURE 1:**
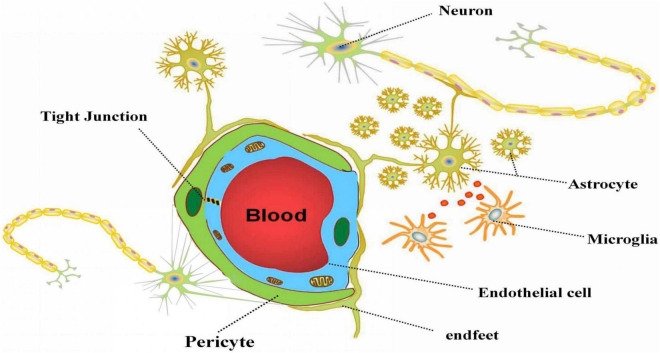
The composition of neurovascular unit. The figure depicts the composition of neurovascular unit and the relationship between the parts in NVU. Neurons, astrocytes, microglia, vascular cells and extracellular matrix together constitute the basic structure of NVU. Astrocytes play a vital role in NVU *via* connect neurons and vascular cells with the end feet, and carry out information transmission with microglia.

Neurovascular unit refers to a dynamic structural complex composed of neurons, glial cells (Ast and MG), vascular cells (endothelial cells, smooth muscle cells, and pericytes), and extracellular matrix (ECM) that maintains the integrity of brain (Naranjo et al., 2021) (as shown in Figure 1). Physiological and pathological stimuli to NVU are affected by a complex molecular interaction between cell-cell, or cell-ECM, even or paracrine cell-cell communication. Intricate signal connections between each element of NVU may affect the whole function of the NVU if any component is damaged (Lo, 2010).

Ast, constituting nearly half of brain cells and outnumbering neurons, are particularly important in the NVU, perform multiple functions to maintain the survival and stability of NVU.

### The Interaction Between Astrocytes and Neurons

Astrocytes, five times of neurons in number, play an important role in neuronal nutrition, support, protection and mutual signal transduction. On one hand, the protrusions of Ast surround the neurons and communicate with neurons and synapses. On the other hand, Ast wraps the blood vessels with the end feet and mediates the transmission of the biological information between neurons and blood vessels. Ast can not only regulate the transmission and strength of synapses, but also participate in the synaptic formation and neuronal differentiation (Stogsdill et al., 2017). Studies have shown that estrogen released by cultured primary Ast increased the number of newly formed synapses and enhanced synaptic signal transmission by activating the estrogen receptor-α on neurons (Fester and Rune, 2015). Thrombospondins-1/2 secreted by Ast promote the synaptic formation in the central nervous system (CNS) both *in vivo* and *in vitro* (Kim et al., 2017). Cholesterol released by Ast promotes synaptic formation and affects the synapse number during the development of neuron (Mauch et al., 2001). The most interesting is that rat embryonic stem cells could differentiate into neurons only in the medium containing Ast (Nakayama et al., 2003). In addition, Ast can provide energy and nutrients for neurons, and release citrate, an important substance supplying neuronal energy during hypoglycemia (Meshitsuka and Aremu, 2008) and produce lactic acid through glycolysis to provide some energy to neurons (Takahashi, 2020).

### The Interaction Between Astrocytes and Microglia

As the innate immune cells in the brain, MG monitor the brain microenvironment, serving as the first line of the CNS defense (Li and Barres, 2018). Generally, MG and Ast are activated into two states: the neurontoxin phenotype (M1/A1) and the neuroprotection phenotype (M2/A2), corresponding to either the destructive or reparative functions in the NVU, respectively (Liu et al., 2020). Activated MG induce A1 polarization of Ast by secreting interleukin (IL)-1α, tumor necrosis factor-α (TNF-α) and complement component 1q (C1q). These cytokines are necessary and sufficient to induce A1 Ast, which lose the ability to promote neuronal survival, outgrowth, synaptogenesis and phagocytosis, inducing the death of neurons and oligodendrocytes (Liddelow et al., 2017). Similarly, MG can also release inflammatory factors such as IL-1β to block the production of sonic hedgehog by Asc, but to drive the secretion of CXCL2 and CCL2, further disrupting the integrity of the BBB and increase neuroinflammatory responses. Ast can release a large amount of IL-17A after stroke, and at the same time, IL-17AR is also highly expressed, and the transformation of MG to M1 type can be reduced by inhibiting IL-17A derived from Ast, which suggests that the polarization of M1-type microglia may be mediated by the IL-17 signaling and is closely related to Ast (Ronaldson and Davis, 2020). MG and Ast can also interact in a paracrine manner by secreting cytokines such as transforming growth factor-β (TGF-β), IL-1, ATP, etc. (Liu et al., 2011). Moreover, a specific pyrimidine-receptor related pathway between MG and Ast, may affect chronic inflammation of the nerve and reaction of glia (Quintas et al., 2014). In addition, MG also affect Ast in the NVU upon stimulation by LPS, whereby MG are activated to produce NO, which subsequently induces neuroprotective effects in Ast through the Keap1/Nrf2 pathway (Takahashi, 2020). MG also release IL-10 to act on reactive astrocytes (RAs), which allow Ast to release cytokines and to play an active protective role in NVU (Zagrean et al., 2018).

### The Interaction Between Astrocytes and BBB

As a complex composed of the brain endothelial cells, pericytes, extracellular matrix basal lamina and astrocytic end-feet (Najjar et al., 2013), BBB is an important structure in the NVU and a natural barrier for the brain to maintain the microvascular homeostasis. Ast, one of the three types of cells involved in the formation of BBB, stretch out to wrap more than 90% of capillary endothelial cells and pericytes, interact with brain microvascular endothelial cells and maintain the integrity of BBB (Thurgur and Pinteaux, 2018). One of main structures of BBB is the endothelial cells and the tight junctions (TJs) between Ast. Ast can express endothelial transport molecules such as glucose transporter-1, and its end-feet can release the signal molecules to support the formation and maintenance of TJs. Inside BBB, Ast are interconnected with neighboring cells through the channels of gap junction that are regulated by extra- and intra-cellular signals and allow exchange of information (Bell et al., 2020). Ast-endothelial gap junctions are mediated by connexin-43 (CX43) hemichannels that allow cell-cell transfer of nutrients, neurotransmitters and maintain water and ion balance inside and outside blood vessels. In this process, there are complex signal exchanges between Ast, pericytes and endothelial cells (Haj-Yasein et al., 2011; He et al., 2013; Nuriya et al., 2013). Prat et al. (2001) found that cultured endothelial cells alone cannot form BBB unless co-cultured with Ast or glial cell culture fluid. Willis et al. (2004) performed a rat model of Ast injury and found that Ast was injured and died at 4–24 h, while BBB permeability increased after 24 h, and then gradually returned to normal at 6–8 days after BBB injury. Therefore, it can be speculated that Ast play an important role in the regulation of BBB permeability. Moreover, interaction between Ast and endothelial progenitor cells (EPCs) may mediate neurovascular remodeling after stroke (Hayakawa et al., 2012).

Pericytes are very important components of the NVU serving as integrators, coordinators and effectors of neurovascular functions, including the maintenance of BBB integrity (Zheng et al., 2020), regulation of cerebral blood flow (CBF) (Kisler et al., 2017a), clearance and phagocytosis of cellular debris (Sá-Pereira et al., 2012). The contraction and relaxation of pericytes play a key role in the regulation of local microvascular blood flow. Some studies have shown that a proportion of pericytes are contractile, responsive to brain-generated vasoactive signals, and are able to influence capillary diameter both *in vitro* and *in vivo* (Hall et al., 2014; Mishra et al., 2016), which can affect the perfusion of local brain and the redistribution of blood flow (Sagare et al., 2013; Kisler et al., 2017b; Montagne et al., 2018). Using two-photon microscopy to observe and manipulate brain capillary pericytes *in vivo*, Hartmann et al. (2021) found that vasoconstriction is associated with a reduction in red blood cell velocity and flux in the same capillary, suggesting that induced contraction of pericytes can alter CBF. As the key cells that regulate the contraction and the relaxation of brain microvasculature, pericytes can affect the formation and permeability of BBB and participate in the reperfusion of blood flow in the brain, which may be, in this sense, closely related to the fact that Ast can affect the Ca^2+^ concentration (Iadecola, 2017). Armulik et al. (2010) found that pericyte deficiency increases the permeability of the BBB, indicating that pericytes play an important role by integrating endothelial cells and Ast, and regulation of the BBB in NVU.

### The Interaction Between Astrocytes and Extracellular Matrix

Extracellular matrix, secreted by neurons or glial cells, surrounding the dynamic structure of cells, provides support for cell arrangement, and performs a series of physiological functions within NVU (De Luca et al., 2020). As one of the components of NVU, ECM can maintain the homeostasis through constant exchanges of water, irons and nutrients in quantity and quality, which are related to the activities of specific enzymes responsible for the degradation of ECM, such as matrix metalloproteinases (MMPs) (Bonnans et al., 2014), Zn^2+^ dependent ECM remodeling endopeptidases (Kucera et al., 2016), playing an important role in the degradation of ECM and the regulation of cell-cell adhesion, migration, infiltration, proliferation, apoptosis (Quattromani et al., 2018), and neuronal synapse remodeling (Oohashi et al., 2015; Stanic et al., 2016; Peng et al., 2018). Studies have confirmed that Ast overexpress MMP-2 and MMP-9 after ischemic stroke, which destroy the structure of ECM and increase the permeability of the BBB (Keep et al., 2014). The main function of MMP-2 is to degrade the basement membrane of the ECM, while the MMP-9 is to degrade and remodel the dynamic balance of the ECM. Min et al. (2017) found that heme can stimulate Ast to release TLR2, then increase the expression of MMP9, causing the damage of ECM and BBB. Some cytokines secreted by Ast can cause changes in the composition and structural integrity of the ECM (De Luca et al., 2018), and conversely, the destruction of the ECM can also significantly influence the function of Ast.

## The Role of Astrocytes in Neurovascular Unit After Ischemic Stroke

**FIGURE 2:**
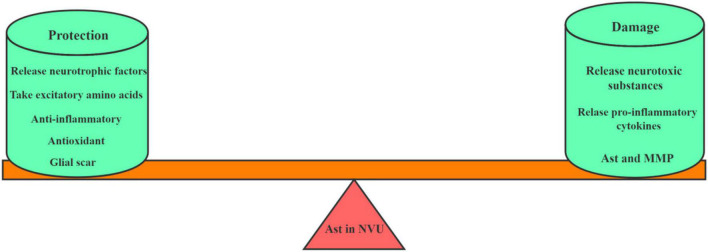
The role of Ast in NVU after ischemic stroke. This picture depicts the dual role of astrocytes in NVU after cerebral ischemia. On the one hand, Ast plays a protective role by secreting neurotrophic factors, taking excitatory amino acids, exerting anti-inflammatory effects and forming glial scars. On the other hand, Ast also plays a damaging role by releasing neurotoxic substances, secreting MMP, and aggravating pro-inflammatory reactions.

Under physiological conditions, Ast secret a variety of neurotrophic factors, such as glial cell-derived neurotrophic factor (GDNF), brain-derived neurotrophic factor (BDNF) and basic fibroblast growth factor (bFGF) at low levels. In the ischemic state, Ast are activated as demonstrated with its change of the morphology, the expression of marker proteins glial fibrillary acidic protein (GFAP) and the increase of vimentin (Pekny et al., 2019). After activated, on one hand, RAs have a strong tolerance under ischemia and protect neurons through various ways, including the release of neurotrophic factors, the uptake of excitatory amino acids, anti-inflammation, antioxidation and the formation of glial scar; on the other hand, they damage neurons by producing excitatory amino acids, activating MG to release inflammatory mediators, and reducing gap junctions, and thus disrupt the BBB. Therefore, Ast are a double-edged sword in the pathological process of cerebral ischemia, which has both neurotoxic and neuroprotective effects on the CNS (as shown in Figure 2).

### Protective Effects

#### The Release of Neurotrophic Factors

Ast are the main source of neurotrophic factors in the CNS. After cerebral ischemia, RAs secrete a variety of nutritional factors, including nerve growth factor (NGF), bFGF, BDNF, GDNF, vascular endothelial growth factor (VEGF) (Liu and Chopp, 2016), erythropoietin (EPO) (Roe, 2017) and neurotrophin-3 (NT-3) (Jurič et al., 2011), which can promote the regeneration of axons and blood vessels, the formation of myelin sheath, and synaptic plasticity. Among them, NGF can antagonize the activities of TNF-α and IL-1β to induce synaptogenesis and inhibit cell apoptosis, therefore exerting neuroprotective functions. bFGF can not only down-regulate the expression of GFAP and other markers to weaken the activation of Ast, but also regulate the upstream TLR4/NF-κB signal pathway to reduce the levels of pro-inflammatory cytokines such as IL-6 and TNF-α (Ye et al., 2015). The injection of bFGF into the lateral ventricle after cerebral ischemia can prevent neuronal damage (Liu et al., 2018). In ischemic stroke, BDNF plays a neuroprotective role in the regulation of signal pathways, the release of cytokines and the inhibition of apoptosis (Li et al., 2021). There is evidence indicating that lower BDNF serum level is associated with increased risk of stroke or transient ischemic attack (TIA) incidence (Pikula et al., 2013). Recent studies have shown that BDNF can also be used as a diagnostic marker for post-stroke complications and a prognostic parameter to predict the functional status of the patients with ischemic stroke (Lasek-Bal et al., 2015; Stanne et al., 2016). GDNF can alleviate learning and memory impairment caused by ischemia *via* promoting cell survival, neurite outgrowth and synapse formation. In addition, GDNF can also regulate Ast proliferation and inflammation in the ischemic penumbra, preventing premature and delayed neuronal death (Steliga et al., 2020). Ast may secrete VEGF, which helps to regenerate microvessels in nerve tissue, increase the density and number of blood vessels and regenerate nerves, and promote the repair of ischemic area. Besides, Ast also produce EPO, which protects hippocampal neurons from ischemic damage by activating Jak2 (Sola et al., 2005). Related studies have shown that EPO can reduce damage to neurons *in vivo* and *in vitro*.

#### The Uptake of Excitatory Amino Acids

Excitotoxic effect is one of the main mechanisms leading to neuronal death during cerebral ischemia. Glutamate (Glu) is mainly produced by Ast and is the main excitatory amino acid in the CNS. However, after the release of Glu, the superfluous Glu in the synaptic cleft can be uptaken by means of glutamate transporter-1 (GLT-1) and glutamate and aspartate transporter (GLAST) to play a neuroprotective effect (Becerra-Calixto and Cardona-Gómez, 2017; Mahmoud et al., 2019). Glu is ingested into Ast and quickly converted to glutamine, which returns to neurons through the glutamate-glutamine cycle (Lau et al., 2010), terminates the signal transduction of excitatory neurons, and maintains low Glu levels. After cerebral ischemia, the ability of RAs to uptake and transform Glu is enhanced, which reduces the accumulation of extracellular Glu, thereby reducing the secondary neurotoxicity caused by excitatory amino acids.

#### Anti-inflammation

Cerebral ischemia usually causes a series of inflammatory reactions, and Ast have a certain anti-inflammatory effect, which can protect the CNS and reduce inflammatory damage. At the peak of inflammation, Ast act as regulatory cells to secrete anti-inflammatory cytokines such as TGF-β and IL-10 to inhibit inflammatory response (Norden et al., 2014). Studies have shown that TGF-β1 is significantly upregulated in the human penumbra region after ischemic stroke. This might be because hypoxia induced stress of neuronal and astroglial cells increases growth factor expression (Krupinski et al., 1996). TGF-β1 upregulates BDNF and ciliary neuronal trophic factor (CNTF) and also induces autosynthesis of TGF-β1 in neurons, which may further explain the existence of the ischemic penumbra, where neurons survive longer because of a protective action of TGF-β1 (Krupinski et al., 1996). Meanwhile, Ast also release lipoxins (Lipoxin A4, LXA4) and (Lipoxin B4, LXB4) to inhibit neuroinflammation, and LXB4 is more effective in neuroprotection than LXA4 (Livne-Bar et al., 2017). Cerebral ischemia and hypoxia may induce the phenotype of A2, which up-regulates a variety of neurotrophic factors and inhibits the spread of inflammatory cells in the CNS to play a protective role in the brain (Li et al., 2019; Jing et al., 2021). In addition, Ast indirectly regulate the function of MG. By inhibiting the MG-mediated inflammatory response, limiting the infiltration of peripheral inflammatory cells and certain bacteria to cross the BBB, Ast repair the damaged BBB, inhibit apoptosis and degranulation of neutrophils, and enhance the phagocytic ability of neutrophils to inhibit the inflammation (Xie et al., 2010). Studies have shown that MG cultured with Ast-conditioned medium increase the expression of their anti-inflammatory gene named CX3CL1 (Li et al., 2018). In addition, Hung et al. (2016) showed that RAs released growth-related protein 43 (GAP-43) *via* the activation of the TLR4/NF-κB/STAT3 signal to inhibit MG and the inflammatory response.

#### Antioxidation

Under pathological conditions such as cerebral ischemia, a large amount of ROS and free radicals will be produced in the brain, causing oxidative damage to cells (Rinaldi et al., 2021). Reduced glutathione (GSH) in the brain is mainly derived from Ast, which scavenges oxygen free radicals, and participates in redox reactions, playing a vital role in anti-oxidative stress (Mashima et al., 2018). Firstly, Ast secrete insulin-like growth factor I and play an anti-oxidative stress effect by activating protein kinase B (Dávila et al., 2016). Secondly, Ast promote NF-E2-related factor 2 (Nrf2) to translocate into the nucleus and activate antioxidant response element (ARE), protecting Ast and adjacent neurons from oxidative damage (Zhou et al., 2015). Thirdly, Ast express Cx43 to form gap junctions counteracting oxidation, and secrete G protein-coupled receptor GPR37-like 1 to protect Ast from oxidative injury (Jolly et al., 2018).

#### The Formation of Glial Scar

After cerebral infarction, the inflammatory response in the brain promotes the activation of Ast and induces the formation of tight junctions of BBB. Meanwhile, RAs around the infarct area secrete extracellular matrix components, especially chondroitin sulfate proteoglycans. Together with the help of microglia/macrophages, glial scars are formed (Huang L. et al., 2014). In this way, a molecular barrier separates the injured area from the surrounding normal tissues, inhibiting the diffusion of toxic substances, and reducing neuronal death caused by excitatory amino acids and neurotoxic substances such as inflammatory mediators (Sato et al., 2008). Studies have found that although glial scars are powerful physical and endocytic barriers, they are unable to completely isolate the liquefied necrotic area from the viable brain tissue, and could not inhibit the neurotoxic factors present in the liquefied necrotic area. This permeability of glial scars may account for the fact that some stroke patients develop neurodegeneration after stroke (Zbesko et al., 2018).

### Damaging Effects

#### The Release of Neurotoxic Substances and Pro-inflammatory Cytokines

Glutamate is an excitatory neurotransmitter. After cerebral ischemia, neurotoxic glutamate accumulates in the intercellular space in a large amount, causing intracellular calcium overloading, and aggravating neuronal damage or death. Ast may secrete a large number of inflammatory mediators such as TNF-α and IL-1β, activating MG to aggravate the degree of cerebral edema, and the inflammatory response of cerebral ischemia (Kadhim et al., 2008; Xin et al., 2021). Thus, activated glial cells, in turn, produce numerous inflammatory mediators which further stimulate the activation and proliferation of glial cells, thereby intensifying the inflammatory response and forming a vicious circle. In this process, TNF-α plays a pivotal role, which induces the release of neurotoxic substances such as glutamate and nitric oxide, and participates in the inflammatory response, while IL-β directly damages neurons.

#### The Secretion of Matrix Metalloproteinases by Astrocytes

Matrix metalloproteinases (MMPs) are composed of a family of zinc-dependent extracellular matrix remodeling endopeptidases (Kucera et al., 2016). In cerebral ischemic diseases, MMPs, secreted by Ast, may not only degrade extracellular matrix, but also destroy the integrity of BBB. Studies have confirmed that there is a certain relationship between acute ischemic stroke and MMPs. Among them, the level of MMP-9 is directly related to the area of cerebral infarction and the hemorrhagic transformation after cerebral ischemia. It was found the serum MMPs levels in the patients with acute ischemic stroke were significantly increased, which aggravated neuronal damage. Knocking out the MMPs gene or adding MMPs inhibitors significantly reduce the damage caused by stroke (Bae et al., 2016). In addition, a variety of drugs targeting MMP-2 and MMP-9 have protective effects on the brain in the mice with cerebral ischemia/reperfusion injury (Zeng et al., 2018).

## Strategies for Targeting Astrocytes in the Treatment of Ischemic Stroke

Ast participate in the pathological process of ischemic stroke by activating or inhibiting a variety of signal pathways, and form a complex molecular signal network to the damaging and repairing process of local NVU at certain stages after ischemic stroke. Given that Ast plays a double-edged sword role on neurons in the process of ischemic stroke, a variety of drugs targeting Ast may have beneficial effects and reduce CNS damage, where ERK1/2 and TLR4/NF-κB pathways are mainly involved. Table 1 lists several drugs or molecules that can potentially target Ast to tackle cerebral ischemia and related mechanisms.

**TABLE 1:** Drugs targeting Ast for ischemic stroke.

Targeting drug	Related regulators and Signaling pathways	Affected processes during cerebral ischemia	References
d,l-PHPB	BDNF, NGF, ERK pathway, PI3K/AKT pathway	Increases the levels of BDNF and NGF secreted by Ast to promote neuronal survival and inhibit neuronal apoptosis by up-regulating ERK and PI3K/AKT signal pathway.	Liu et al., 2017
Tanshinone IIA	HIF-1α, SDF-1, ERK1/2, AKT	Inhibit the accumulation of HIF-1α and stromal cell derived factor-1 in Ast under OGD conditions, the activation of ERK1/2 and AKT, the proliferation of Ast.	Huang X. et al., 2014
Ginkgo diterpene lactones (GDL)	IL-1β, TNF-α, TLR4/NF-κB pathway	GDL attenuates ischemic injury by inhibiting the TLR4/NF-κB pathway, inhibiting platelet aggregation, astrocyte activation and IL-1β and TNF-α production.	Li et al., 2020
Pien Tze Huang (PTH)	IL-1β, IL-6, TNF-α, MCP-1, TLR4, MyD88, TRAF6, NF-κB, p-ERK1/2	PTH reduces the expression of IL-1β, IL-6, TNF-α and MCP-1 by downregulating TLR4, MyD88 and TRAF6 and reducing the expression and nuclear translocation of NF-κB. And reduce the protein expression of p-ERK1/2 to exert neuroprotective effect after ischemia.	Zhang et al., 2021
Fasudil	TNF-α, IL-6, IL-10, IL-4, TLR4/NF-κB pathway	Fasudil inhibits the activation of TLR4/NF-κB signal pathway and inflammatory response, exerting a neuroprotective effect.	Zhang H. et al., 2018
Ginkgolide K (GK)	AMPK/mTOR/ULK1 pathway	GK promoted Ast proliferation and migration after OGD *via* inducing protective autophagy through the AMPK/mTOR/ULK1 signaling pathway.	Zhang and Miao, 2018
Baicalin	BDNF, TrkB, PI3K/AKT, MAPK/ERK1/2	Increasing the release of BDNF and its associated receptor TrkB and downstream signaling regulators PI3K/Akt and MAPK/ERK1/2 against oxidative stress, inflammation, and apoptosis.	Li et al., 2021
Astragaloside IV (AS-IV)	CXCR4, p-JNK/JNK pathway, Bax/Bcl-2, Nrf2/Keap1	Inhibit CXCR4 receptor and downregulated the activation of p-JNK/JNK pathway, which suppressed the expression of Bax/Bcl-2, and finally uprising Nrf2/Keap1 signaling. AS-IV protected against OGD/R-induced Ast through inhibiting oxidative stress and apoptotic pathways.	Yang et al., 2021
Salidroside	Akt/GSK-3β	Inhibit RAs proliferation, ameliorate glial scar formation and improve long-term recovery, probably through its effects on the Akt/GSK-3β pathway.	Dong et al., 2021
Glycyrrhizic acid	HMGB1-TLR4 axis	Attenuated OGD/R-induced impairment of astrocytic glutamate clearance mediated by the HMGB1-TLR4 axis.	Lin et al., 2020

### ERK1/2 Signal Pathway

ERK1/2 is a key regulator in the MARK signal pathway and plays an important role in cell proliferation and differentiation. Studies have shown that activation of the ERK1/2 signal pathway also has dual effects to ischemic stroke (Maddahi and Edvinsson, 2010). On one hand, inflammatory factors or oxidative stress activates ERK1/2, which aggravates NVU damage; on the other hand, after the activation of ERK1/2 signal pathway, neurotrophic factors secreted by Ast play a neuroprotective role (Sawe et al., 2008). Some drugs targeting Ast can affect ischemic stroke through the ERK1/2 pathway. For example, Potassium2-(1-hydroxypentyl)-benzoate (D, L-PHPB), a new drug candidate for ischemic stroke at the phase II clinic trial. By up-regulating ERK activity, D, L-PHPB significantly increases the levels of BDNF and NGF secreted by Ast to promote neuronal survival and inhibit neuronal apoptosis under OGD/R conditions in the co-culture system of neurons and Ast (Liu et al., 2017). D, L-PHPB treatment suggests that the neuroprotective effects may be related to Ast (Zhang et al., 2006; Hu et al., 2012; Zhao et al., 2013; Peng et al., 2014), but the mechanisms of D, L-PHPB remain to be further studied. Tanshinone IIA inhibits the accumulation of hypoxia inducible factor-1α and stromal cell derived factor-1 in Ast under OGD conditions, the activation of ERK1/2 and AKT, and the proliferation of Ast (Huang X. et al., 2014), thereby reducing the degree of injury.

### TLR4/NF-κB Signal Pathway

TLR4 is the earliest discovered Toll-like protein. After cerebral ischemia, TLR4 is firstly activated in both Ast and MG. NF-κB is one of the important downstream signaling nuclear transcription factors of TLR4. Some cytokines such as IL-2, IL-1β and TNF-α in the cell can activate the NF-κB in turn, thereby stimulating further cascade amplification of inflammatory signals. Inhibiting the activation of the TLR4/NF-κB signal pathway improves the damage of ischemic stroke (Zhao et al., 2018). Studies have shown that ginkgo diterpene lactones attenuates ischemic injury by inhibiting the TLR4/NF-κB pathway, inhibiting platelet aggregation, astrocyte activation and pro-inflammatory cytokine production (Li et al., 2020). Pien Tze Huang (PTH) significantly reduces cerebral infarct volume in MCAO rats, improves neurological function scores and pathological damage, which may be related to reducing the expression of IL-1b, IL-6, TNF-α and MCP-1 by downregulating TLR4, MyD88 and TRAF6 and reducing the expression and nuclear translocation of NF-κB. In addition, PTH can also reduce the levels of p-ERK1/2 to exert neuroprotective effect after ischemia (Zhang et al., 2021). Fasudil inhibits the activation of TLR4/NF-κB signal pathway and inflammatory response, exerting a neuroprotective effect (Zhang H. et al., 2018). In addition, Enriched environment (EE) is a method of exogenous intervention on the nervous system through behavioral intervention, which promotes neurogenesis and functional recovery through NF-kB-mediated secretion of IL-17A by Ast in stroke patients (Zhang Y. et al., 2018).

## Summary and Prospects

In summary, Ast, an important component in the NVU and closely connected to neurons, BBB and other glial cells, are activated in ischemic stroke and play an crucial double-edged sword role. On one hand, Ast play a protective role through anti-inflammatory, antioxidant, and release of neurotrophic factors. On the other hand, Ast release neurotoxic substances that damage BBB and increase the area of cerebral infarction. Various signal pathways form a complex signaling molecular network in the NVU, and Ast participate in this exquisite network and play an important regulatory role.

At present, there are still many difficulties in the treatment of ischemic stroke, and the interaction between signal molecules needs to be further elucidated. Although a variety of drugs have shown that targeting Ast has a certain positive effect on recovery after stroke, due to the complexity and diversity of the underlying mechanisms, most drugs are still in experimental research or phase I and phase II clinical trials. Given that Ast play a dual role in the integrity of the NVU, addressing homeostasis of Ast would be critical to tackle ischemic stroke-related conditions. Targeting Ast to treat ischemic stroke needs continuous attentions in the future.

## Author Contributions

GH, LS, and QW outlined and drafted the manuscript. CM helped coordinate and draft the manuscript. ZD and YY were involved in the revisions. CM and JH revised and finalized the manuscript. All authors read and approved the final manuscript.

## Conflict of Interest

The authors declare that the research was conducted in the absence of any commercial or financial relationships that could be construed as a potential conflict of interest.

## Publisher’s Note

All claims expressed in this article are solely those of the authors and do not necessarily represent those of their affiliated organizations, or those of the publisher, the editors and the reviewers. Any product that may be evaluated in this article, or claim that may be made by its manufacturer, is not guaranteed or endorsed by the publisher.
